# Evaluation of Microleakage Using Different Luting Cements in Kedo Zirconia Crowns: An In Vitro Assessment

**DOI:** 10.7759/cureus.66237

**Published:** 2024-08-05

**Authors:** Guru Vishnu, Ganesh Jeevanandan

**Affiliations:** 1 Department of Pedodontics and Preventive Dentistry, Saveetha Dental College and Hospitals, Saveetha Institute of Medical and Technical Sciences, Saveetha University, Chennai, IND

**Keywords:** crowns, primary molars, luting cement, thermocycling, milk teeth

## Abstract

Introduction

A space between the prepared tooth and the cemented crown can cause microleakage, allowing microorganisms to enter, which in turn leads to the breakdown of the luting cement. To achieve an optimum fit, several factors should be considered, including the type of crown used, the taper of tooth preparation, and the type of cementing agent.

Aim

The purpose of the current study is to evaluate the microleakage of zirconia crowns cemented with glass ionomer cement (GIC), resin-modified GIC (RMGIC), and self-adhesive resin cement.

Materials

Forty-five primary mandibular molars were divided into three groups: Group I receiving GIC, Group II receiving RMGIC, and Group III receiving self-adhesive resin cement. These cements were used to lute the teeth with Kedo zirconia crowns. The restored samples underwent thermal cycling and were assessed for microleakage under a stereomicroscope. For the statistical analysis, SPSS version 23.0 was used. Descriptive statistics were presented as frequencies and percentages. Analytical statistics, including the Mann Whitney U test, were used to assess the differences in the level of microleakage between the groups at p < 0.05.

Results

The Mann Whitney U test revealed no significant difference in the level of microleakage between GIC and RMGIC (p = 0.072). However, self-adhesive resin cement showed significantly less microleakage than GIC (p = 0.000). Similarly, when comparing RMGIC and self-adhesive resin cement, the latter showed significantly less microleakage than the former (p = 0.001).

Conclusion

Microleakage of Kedo zirconia crowns on mandibular first molars was highest when luted with GIC, followed by RMGIC, and was least when luted with self-adhesive resin cement.

## Introduction

The past 10 years have been a standout period for dental material development, particularly with the rapid rise in metal-free dentistry and the use of zirconia materials, which offer great biocompatibility, superior material strength, and enhanced aesthetics. A detailed understanding of the chemistry, crystallography, and tailored ceramic properties of zirconia ceramics has resulted in their enhanced application in dentistry [1]. Zirconia, a crystalline zirconium dioxide, resembles the color of teeth and possesses mechanical properties akin to metals. Both primary incisors and molars can now be fitted with pre-made zirconia crowns [2]. Studies on monolithic ceramic and metal-ceramic restorations show that zirconia crowns have durability and survival rates comparable to those of stainless steel crowns [3,4]. Inadequate marginal fit can lead to plaque accumulation, dental caries, cement disintegration, secondary caries, dentin hypersensitivity, and periodontal inflammation [5].

For zirconia, luting cements come in various forms, including zinc phosphate, polycarboxylate, glass ionomer, resin cement, and bioactive cement [6]. However, there are no set guidelines for cementing the crown. Previous studies include one by Samuel Raj Srinivasan SR et al., where different luting cements were compared in a 36-month follow-up for NuSmile Zirconia crowns [7], and another by Azab MM et al., which examined the influence of luting cement on the clinical outcomes of zirconia pediatric crowns in a three-year split-mouth study, concluding that glass ionomer cement (GIC) performed better than bioactive cement [8]. However, there have been no studies on Kedo Zirconia Crowns where microleakage was assessed using different luting cements.

The clinical and material-based flaws prevent us from achieving a correct marginal fit, which causes microleakage of the luted cement [9]. Some authors argue that there is a correlation between marginal disparity and micro-infiltration [10]. It is important to note that the durability of the restoration depends on the cementing agent, making the properties of the cement crucial for preventing microleakage and achieving an appropriate marginal fit [11]. According to a study by Jacob J et al., zinc phosphate cement is less efficient than glass-ionomer and resin cements in minimizing microleakage [12]. This inefficiency may be due to the higher solubility of zinc phosphate cements, which rely solely on a mechanical bond, compared to glass-ionomer and resin cements [13,14]. Similarly, increasing the axial convergence angle of the preparation should improve the marginal fit [15]. Absolute marginal discrepancy (AMD) is the preferred metric for determining vertical and horizontal marginal mismatch. However, standardizing marginal discrepancy remains challenging. Microleakage is measured using both invasive (by sectioning the tooth after dye penetration) and non-invasive (by using the direct view technique) methods. The invasive method is considered more precise because it allows for accurate observation of dye penetration using various chromatic solutions such as methylene blue, safranin, and fuchsine. This observation is performed under a stereomicroscope using the intrusive approach, which was employed in this experiment [12]. The goal of this study is to assess the microleakage of zirconia crowns luted with resin-modified glass ionomer cement (RMGIC), self-adhesive resin cement (SARC), and GIC [16].

Chang B et al. previously conducted measurements of microleakage around zirconia crown margins that were secured using RMGIC after ultrasonic scaling [17]. Ebadian B et al. conducted an additional in vitro experiment to evaluate the influence of different luting cements and the angle of tooth preparation on the extent of microleakage in zirconia crowns placed on permanent teeth [18]. There have been no previous studies comparing the microleakage of different luting cements on Kedo Zirconia crowns.

## Materials and methods

The present study is an in-vitro study that was carried out at White Lab, Saveetha Dental College, Chennai from September to October 2023 after a scientific review board number was obtained. The scientific review board granted approval for the methodology of the study (SRB/SDC/PEDO-2105/23/159).

The inclusion criteria were extracted human primary mandibular first molars, with or without dental caries. Human teeth without any fractures or craze lines were included in the study. Teeth with proximal caries were also included. The exclusion criteria were that human teeth with incomplete root formation were not used. Teeth with a high rate of root resorption were excluded from the study. Caries on the buccal or lingual surfaces of the teeth were omitted. The power of the sample size was determined based on the study by Ebadian B et al. conducted in 2021 [16], having a p-value of 0.05 and a power of 95%, the study detected an effect size of 0.124. Forty-five samples made up our determined sample size.

A total of forty-five teeth were collected and subjected to surface debridement using hand scalers to eliminate soft tissues and calculus. Three sets of teeth were evenly split among the groups: Group I receiving GIC for luting, Group II receiving RMGIC for luting, and Group III receiving SARC for luting the zirconia crowns. The 45 primary human first molars used in the study were either healthy or had caries with a root resorption rate under two-thirds. The teeth were immersed in distilled water and kept at a constant temperature of 23°C for a maximum duration of three weeks. The study excluded teeth that had lingual or buccal caries. Caries-free buccal and lingual surfaces were left intact after cleaning carious teeth. For teeth with pulp exposure, pulp remains were eliminated, and GIC was used to fill the pulp chamber after a seven-second etch-and-rinse procedure (Eco-Etch, Ivoclar, Switzerland) and bonding (Vivadent Te-econom Bond, Ivoclar, Switzerland). The cavity was filled with composite resin (Te Econom Plus Composite, Ivoclar, Switzerland) and returned to its former shape. The teeth were randomly separated into three groups (A, B, and C), with 15 teeth in each group. They were subsequently anchored in transparent cold-cure acrylic resin blocks, ensuring that the blocks extended up to 2 mm below the cement-enamel junction. This embedding process took place during the 40-second polymerization stages for both the glue and each layer of the resin composite.

Specimen preparation

The same operator handled all tooth preparations. The teeth that received Kedo zirconia crowns underwent standardized dental preparations in accordance with the manufacturer's recommendations. The occlusal surfaces of the teeth in each group were roughened by 1-1.5 mm using a coarse football diamond bur (FO-27, Mani, Japan). Reductions of 0.5 mm were achieved on the mesial, distal, lingual, and buccal surfaces using a coarse tapered diamond bur (TR-S21, Mani, Japan). Before the luting cements were used to cement the crowns, the preparation margin was checked with a probe for undercuts, polished to a feather-edge on all surfaces, and all line angles were rounded (TR-21EF, Mani, Japan). The teeth were randomly assigned to each group (n=15 per group), a luting cement was assigned to each group, and the exact crown sizes were meticulously calculated. Group I received GIC (37200, 3M™ Ketac Cem, USA), Group II received RMGIC (3525TKA, 3M™ RelyX™ Luting RMGIC, USA), and Group III received SARC (Rely XTM, 3M ESPE, USA). According to the directions provided by the manufacturer, the luting cements were mixed and then the crowns were luted. The crowns in each group were applied to the preparations with finger pressure after being filled with the appropriate luting cement. To ensure the stability of the crowns while the cement was hardening, a weight of 5 kg was applied axially to the crowns using a loading device for a duration of 10 minutes after the cement mixing started.

Assessment of microleakage

The samples were preserved for 24 hours in distilled water. The teeth were then subjected to thermocycling. The useful ranges of temperature were 12°C + 2 and 60°C + 2. One minute was utilized to immerse samples alternately in hot and cold solutions. The restorations underwent a total of 1500 cycles, simulating a period of 6 months within the patient's oral cavity. All tooth surfaces, with the exception of a 1 mm border surrounding the restoration, received two coats of nail polish. Sticky wax was used to seal the apices. The samples were submerged in a solution containing 2% methylene blue dye for a duration of 24 hours. Subsequently, the teeth were rinsed with distilled water and dried. After that, the restorations were split in half mesiodistally to make two test specimens from each tooth, exposing the tooth-to-tooth contact from the cavosurface margin to the pulpal wall. The specimens were analyzed using a stereomicroscope at a 10x magnification. The extent of marginal leakage was evaluated based on the criteria established by Khera and Chan [19]:

0º= Microleakage at crown margins only

1º= Microleakage at crown margins and around cement

2º= Microleakage at crown margins and throughout cement

3º= Microleakage to 1/3 of tooth structure

4º= Microleakage throughout tooth structure and pulp

Statistical analysis

The data was entered into a Microsoft Excel spreadsheet and then analyzed using SPSS software (version 23.0). The data was analyzed using descriptive statistics, which included measures such as percentages, frequency, SD, mean, and a 95% confidence interval. The Shapiro-Wilk test was used to assess the normality of the distribution of all parameters. Descriptive data were represented by the mean and SD. The levels of microleakage in the groups were compared using the Mann-Whitney U test to see if there was a significant difference at p<0.05.

## Results

The current investigation comprised 45 samples categorized into three groups: GIC, RMGIC, and SARC. The data underwent normality analysis using the Shapiro-Wilk test, which demonstrated that the microleakage for all groups had a normal distribution. Among all the samples tested, the highest degree of microleakage was noticed with GIC, followed by RMGIC, with the least degree of microleakage noticed with SARC.

Among the 15 samples in the GIC group, 0 (0%) samples showed zero degrees of microleakage, 6 (40%) samples showed first-degree microleakage, 5 (33.3%) samples showed second-degree microleakage, 3 (20%) samples showed third-degree microleakage, and 1 (6.7%) sample showed fourth-degree microleakage. Among the 15 samples in the RMGIC group, 2 (13.3%) samples showed zero degrees of microleakage, 7 (46.7%) samples showed first-degree microleakage, and 6 (40%) samples showed second-degree microleakage. Among the 15 samples in the SARC group, 10 (66.7%) samples showed zero degrees of microleakage, and 5 (33.3%) samples showed first-degree microleakage. Table 1 displays the distribution of microleakage values for the three groups.

**Table 1:** Distribution of the degree of microleakage among the study groups.

Degree of microleakage	Glass ionomer cement		Resin-modified glass ionomer cement		Self-adhesive resin cement	
	N	%	N	%	N	%
0	0	0	2	13.3	10	66.7
1	6	40	7	46.7	5	33.3
2	5	33.3	6	40	0	0
3	3	20	0	0	0	0
4	1	6.7	0	0	0	0

**Figure 1:**
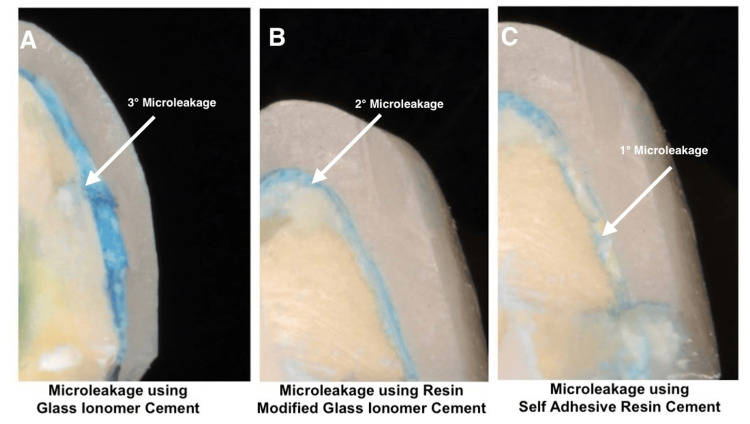
Assessment of microleakage using various luting agents: A - Using glass ionomer cement; B - Using resin-modified glass ionomer cement; C - Using self-adhesive resin cement.

Figure 1 displays the degree of microleakage for one sample out of the total of 15 samples collected for each group.

Figure 1A is a representative stereomicroscopic image of a sample that was bonded using GIC, resulting in a third degree of microleakage covering up to one-third of the tooth structure. Figure 1B is a representative stereomicroscopic image of a sample that was bonded using RMGIC, resulting in a second degree of microleakage at the crown and throughout the cement. Figure 1C is a representative stereomicroscopic image of a sample that was bonded using SARC, resulting in first-degree microleakage at the crown margin and around the cement. Thus, it is evident that SARC exhibits the lowest level of microleakage, followed by RMGIC, while GIC has the highest degree of microleakage.

There was no statistically significant difference between the levels of microleakage in GIC and RMGIC cements (p=0.072), according to the Mann-Whitney U test, which is used to measure the mean difference of microleakage pairwise. However, a comparison of GIC and Self-adhesive resin cement (p=0.000*) revealed that the latter had a statistically significant difference in the degree of microleakage. Self-adhesive resin cement demonstrated much less microleakage than RMGIC, with a p-value of 0.05 (Table 2).

**Table 2:** Mann-Whitney U tests were performed pairwise to assess the differences in the level of microleakage among the GIC, RMGIC, and SARC groups. GIC: Glass ionomer cement; RMGIC: Resin-modified glass ionomer, SARC: Self-adhesive resin cement. *: p<0.05.

Groups for comparison	Study groups	Mean rank	Sum of ranks	Mann-Whitney U value	P-value
GIC vs RMGIC	GIC	18.2	273	72	0.072
	RMGIC	12.8	192		
GIC vs SARC	GIC	22	330	15	0.000*
	SARC	9	135		
RMGIC vs SARC	RMGIC	20.5	307.5	37.5	0.001*
	SARC	10.5	157.5		

## Discussion

The invention of RMGICs represents a notable advancement in glass ionomer technology that has positively impacted pediatric dentistry. The glass ionomer and resin constitute only a small portion of the RMGICs. The initial hardening of RMGICs occurs as a result of the resin component being photopolymerized by free radicals [9,11]. This is followed by a chemical setting process of the glass ionomer. Adding the resin component not only reduces handling challenges and initial hardening times but also significantly enhances the cement's physical properties and wear resistance. Resin luting systems are also recommended for the cementation of all-ceramic systems. Bernard et al. reported that significantly stronger all-ceramic crowns can be achieved by luting with adhesive resin cements [20].

Zirconia crowns are frequently luted with GIC because it provides several therapeutic benefits, including long-term fluoride release, low coefficients of thermal expansion, and physicochemical attachment to tooth structures [21,22]. Resin cement is composed of bisphenol-A-glycidyl methacrylate and other methacrylates, featuring strong compressive strength, low solubility, and high bonding strength [21]. Due to the shortcomings of other cements, including their lack of solubility, support, and adherence, resin cement is often used as the preferred option for ceramic restorations [23], significantly contributing to the ultimate clinical success of the treatment. SARCs are typically dual-cured resins that can be light-activated and can self-cure [24]. They are esthetically pleasing and exhibit less microleakage compared to other cements. SARCs offer improved properties and less technique sensitivity than traditional cements. The etch-and-rinse adhesive resin cement exhibits strong etching and resin infiltration, leading to the creation of a hybrid layer. This process results in stronger micromechanical bonding to the tooth structure and, consequently, the lowest microleakage scores [25,26]. In the present study, 40% of the samples with GIC cements exhibited first-degree microleakage. In samples with RMGIC, 13.3% had no microleakage. Similarly, in a study conducted by Pontes DG et al., RMGIC showed less microleakage than conventional GIC [27]. In a study by Rekha CV et al., 15.6% of samples with GIC had no leakage, whereas 82.8% of samples with RMGIC exhibited no microleakage at all [28]. Another study by Yüksel E and Zaimoğlu A noted a significant difference in the amount of microleakage between GIC and SARC, with GIC exhibiting higher microleakage [29]. The present analysis revealed that GIC had the highest level of microleakage, followed by RMGIC, and then self-adhesive resin cement. In research conducted by Al-Haj Ali SN et al., resin cement showed statistically significant differences from GIC in having the least microleakage [30]. Similarly, another study found that RMGIC showed a higher degree of microleakage than self-adhesive resin [17].

This study had a few limitations that could have exaggerated the observed degree of microleakage. There were discrepancies in the depth of cavity preparation, with different measurements tailored to the needs of each tooth. Since all specimens were bonded outside of the oral cavity, each step was completely visible, and the edges were accessible from all sides. These in-vitro conditions enable practically flawless margin preparation, optimal tooth margin, and full access for removing excess cement. As a result, a virtually flawless fit was possible in most cases, with all of the cement flush with the tooth and crown. However, in therapeutic settings, there might be spots with open edges, cement gaps dug too deep, or poor bonding. These locations might be more vulnerable to deterioration from future ultrasonic scaling or masticatory forces.

## Conclusions

Considering the limitations of the investigation, it was found that GIC and RMGIC exhibited the highest rates of microleakage, closely followed by SARC. No significant differences were observed between GIC and RMGIC, suggesting their similar performance in preventing microleakage. However, SARC demonstrated substantial and noteworthy differences, performing better than both GIC and RMGIC. This highlights the superior adhesive properties and sealing ability of SARC, making it a more reliable option for minimizing microleakage in dental restorations.
